# Soil fungal communities of ectomycorrhizal dominated woodlands across West Africa

**DOI:** 10.3897/mycokeys.81.66249

**Published:** 2021-06-11

**Authors:** Peter Meidl, Brendan Furneaux, Kassim I. Tchan, Kerri Kluting, Martin Ryberg, Marie-Laure Guissou, Bakary Soro, Aïssata Traoré, Gbamon Konomou, Nourou S. Yorou, Anna Rosling

**Affiliations:** 1 Department of Ecology and Genetics, Evolutionary Biology, Uppsala University, Norbyvägen 18D, Uppsala 752 36, Sweden; 2 Department of Organismal Biology, Systematic Biology, Uppsala University, Norbyvägen 18D, Uppsala 752 36, Sweden; 3 Research Unit in Tropical Mycology and Plant-Soil Fungi Interactions, LEB, University of Parakou, Parakou, Benin; 4 Université Norbert Zongo, Unité de Formation et de Recherches en Sciences et technologies. BP 376 Koudougou, Burkina Faso; 5 Université Nangui Abrogoua, URF Sciences de la Nature, Laboratoire d’écologie et de Développement Durable, Abidjan, Ivory Coast; 6 Institut d’Economie Rurale (IER), Rue Mohammed V, Bozola, BP 258, Bamako, Mali; 7 Herbier National de Guinée / Université Gamal Abdel Nasser de Conakry BP: 1147, Conakry, Guinée

**Keywords:** biodiversity, eDNA, fungal community, gallery forest, Guineo-Sudanian woodlands

## Abstract

Forests and woodlands in the West African Guineo-Sudanian transition zone contain many tree species that form symbiotic interactions with ectomycorrhizal (ECM) fungi. These fungi facilitate plant growth by increasing nutrient and water uptake and include many fruiting body-forming fungi, including some edible mushrooms. Despite their importance for ecosystem functioning and anthropogenic use, diversity and distribution of ECM fungi is severely under-documented in West Africa. We conducted a broad regional sampling across five West African countries using soil eDNA to characterize the ECM as well as the total soil fungal community in gallery forests and savanna woodlands dominated by ECM host tree species. We subsequently sequenced the entire ITS region and much of the LSU region to infer a phylogeny for all detected soil fungal species. Utilizing a long read sequencing approach allows for higher taxonomic resolution by using the full ITS region, while the highly conserved LSU gene allows for a more accurate higher-level assignment of species hypotheses, including species without ITS-based taxonomy assignments. We detect no overall difference in species richness between gallery forests and woodlands. However, additional gallery forest plots and more samples per plot would have been needed to firmly conclude this pattern. Based on both abundance and richness, species from the families Russulaceae and Inocybaceae dominate the ECM fungal soil communities across both vegetation types. The community structure of both total soil fungi and ECM fungi was significantly influenced by vegetation types and showed strong correlation within plots. However, we found no significant difference in fungal community structure between samples collected adjacent to different host tree species within each plot. We conclude that within plots, the fungal community is structured more by the overall ECM host plant community than by the species of the individual host tree that each sample was collected from.

## Introduction

Throughout West Africa, forests, woodlands and savannas represent ecosystems of great biodiversity and economic importance as resources for food, fuel and fiber (Sinsin and Kampmann 2010). In many parts of the region, forest land is under pressure from grazing, conversion to cropland, fuelwood extraction and replacement by plantations of non-native trees, often resulting in fragmentation of the landscape (Assédé et al. 2020). These threatened ecosystems harbor a tremendous undescribed diversity of fungi, a group of organisms that is particularly understudied in tropical regions, and particularly so in West Africa (Gryzenhout et al. 2012; Piepenbring et al. 2020). Ectomycorrhizal (ECM) symbiosis is a mutualistic relationship in which fungal hyphae surround and grow between the cortical cells of specialized fine plant roots. The relationship benefits both organisms, with the fungal partner providing nutrients in exchange for carbon from the plant partner. Although only certain groups of fungi and plants can form ECM, this capacity has evolved convergently in around 80 fungal lineages (Tedersoo and Smith 2017) and 30 plant lineages (Tedersoo and Brundrett 2017), the latter of which are often dominant trees in forests and woodlands. ECM fungi play an important role for plant health and forest regeneration by facilitating nutrient and water uptake to their host plants. Globally, ECM fungi represent about 8% of described fungal species (Ainsworth 2008; Rinaldi et al. 2008; Hawksworth and Lücking 2017). Despite their importance both in nature and the anthropogenic world, fungal diversity remains poorly characterized, particularly in tropical regions (Tedersoo et al. 2014). Increased knowledge about existing fungal biodiversity of wooded ecosystems is an important step towards building sustainable land use management that balances production and conservation.

**Figure 1.**
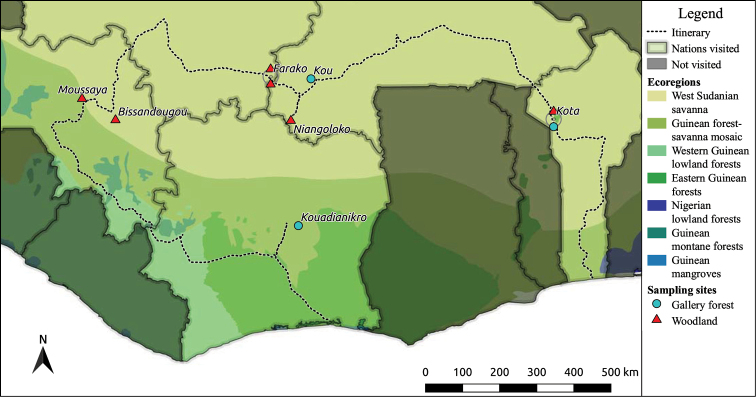
Sampling sites of the West African Centre for Tropical Mycology’s 2018 National Geographic Explorer Grant expedition. Shapes and colors separate the different woodland types with blue circles for gallery forests and red triangles for woodlands. With site names (abbreviations): Bissandougou (BISS-W), Moussaya (MOUS-W), Kota (KOTA-G and KOTA-W), Kouadianikro (KDNK-W), Kou (KOUF-G), Niangoloko (NIAN-W) and Farako (FA01-W and FA15-W). The dotted line represents the route taken on the sampling trip, beginning on the coast of Benin and concluding in Ivory Coast. Ecoregions are from White (1983), digitized in Olson et al. (2001).

Wooded vegetation covers much of West Africa south of the Sahelian savanna and is geographically structured largely based on water availability (Swaine 1992). In the West Sudanian savanna and Guinean forest-savanna mosaic ecoregions, which together form the Guineo-Sudanian transition zone (Fig. 1), woodland growth is water limited due to seasonal drought, and canopy coverage is typically between 15–40% (Swaine 1992; Paré et al. 2008). Drought and fire are important factors determining plant community structure in these woodlands (Savadogo 2007). Characteristic Guineo-Sudanian woodland trees (Adomou 2005; Assédé et al. 2020) include species in the genera *Isoberlinia*, *Afzelia* and *Anthonotha* (Fabaceae), *Uapaca* (Phyllanthaceae) and *Monotes* (Dipterocarpaceae), all of which form symbiotic associations with ECM fungi (Yorou et al. 2014; Tedersoo and Brundrett 2017; Houdanon et al. 2019). Gallery forests are another characteristic vegetation type in the region. Gallery forests form narrow corridors in riparian areas (i.e., along rivers), typically in areas which are too dry for closed-canopy forest to grow outside of the riparian area (Natta et al. 2003). Thus, the gallery forest edge is distinct in relation to surrounding habitat types (Paré et al. 2008). While only a small proportion of African tree species form ECM associations, the total number of tree species is much higher than in boreal and temperate regions (Bâ et al. 2012). Despite this, Africa has the highest number of tree species forming associations with ECM fungi of all tropical regions, and these ecosystems are often characterized by the dominance or co-dominance of ECM trees such that these trees represent a critical ecological component (Corrales et al. 2018). The occurrence of stands of predominantly ECM forming tree species (Houdanon et al. 2019) has led to the hypothesis that ECM fungi facilitate establishment of other ECM seedlings (Corrales et al. 2018). Further, low levels of host specificity have been demonstrated for ECM fungi in Africa, potentially allowing for the formation of common ECM networks linking different host species below ground (Diédhiou et al. 2010; Tedersoo et al. 2011).

Fungal diversity in West Africa is understudied and only recently was a fungal check list for West Africa completed to facilitate monitoring and communication of fungal diversity (Piepenbring et al. 2020). The checklist is an important first step to assess the role and prevalence of fungal diversity in West Africa (Yorou and De Kesel 2011). The checklist contains only already described fruiting body-forming fungi, which represent only a fraction of the estimated fungal diversity (Hawksworth and Lücking 2017). In fact, global estimates based on environmental sequencing indicate that the majority of extant soil fungi remain undescribed (Tedersoo et al. 2017).

The recent proliferation of environmental DNA-based studies has overcome many limitations of fruiting body-based surveys, advancing knowledge of large-scale patterns of fungal diversity (Sato et al. 2012; Tedersoo et al. 2014; Barberán et al. 2015; Davison et al. 2015). Molecular identification of fungi from soil and roots has greatly improved our ability to characterize ECM fungal communities independently from fruiting bodies, whose formation varies with various temporal and spatial factors. Such studies suggest that, contrary to biodiversity patterns of animals and plants, ECM fungi decrease in species richness towards the equator, with lower diversity in tropical compared to temperate forests (Tedersoo and Nara 2010). In West Africa, ECM fungal communities are dominated by species in the four families Russulaceae, Thelephoraceae, Boletaceae and Sclerodermataceae (Bâ et al. 2012; Tedersoo and Smith 2013, 2017). Species in the Amanitaceae are also widespread throughout the tropics (Corrales et al. 2018). Surveys based on fruiting bodies confirm this pattern, reporting numerous members of these families (Ducousso et al. 2003; Yorou 2008; Bâ et al. 2011; Sanon et al. 2014; Yorou et al. 2016; Piepenbring et al. 2020).

Although woodlands and gallery forests in the Guineo-Sudanian transition zone are known to host many ECM fungi, little is known about how ECM tree composition and density affect the abundance and composition of ECM and other fungi in soil. The majority of existing studies describing fungal biodiversity in West Africa rely on observation and collection of fruiting bodies. Because these structures are highly ephemeral, and many species don’t produce them at all, sequencing DNA from soil samples is a more reliable means of providing a more complete perspective of a given soil fungal community. As ECM-dominated vegetation in West Africa varies widely in tree species composition and structure (Houdanon et al. 2019), we suspect similar differences in the below-ground fungal community. To capture the regional diversity of ECM fungi, we collected soil samples in vegetation dominated by ECM host trees across the five West African countries Benin, Burkina Faso, Mali, Guinea and the Ivory Coast. We leveraged the capability of the PacBio Sequel system to provide high quality reads of over 1 kb in length to sequence the full internal transcribed spacer (ITS) and partial large subunit (LSU) of the nuclear ribosomal DNA. This allowed a model-based approach to generating species hypotheses, based on a phylogenetic tree generated from the more conserved LSU region, as well as a combined approach to taxonomic identification involving both similarity-based assignment on the ITS region and reference to the LSU-based phylogenetic tree. We analysed how ECM host tree species community structure, total soil fungal communities and ECM fungal communities varied in two different vegetation types. Our data provide an important baseline resource for future biodiversity studies of soil fungal communities in West Africa.

## Methods

### Field site characteristics and soil sampling

Field collections were carried out during June and July of 2018 in five West African countries: Benin, Burkina Faso, Mali, Guinea and Ivory Coast. Sites were selected opportunistically from natural areas where ECM host trees were present, with relatively uniform vegetation and slope. A total of seven locations with nine sites were sampled: Kota Waterfall (KOTA-G and KOTA-W) in Benin, Kou Forest Reserve (KOUF-G) and Niangoloko Forest Reserve (NIAN-W) in Burkina Faso, Farako Forest Reserve (FA01-W and FA15-W) in Mali, Bissandougou Forest Reserve (BISS-W) and Moussaya Forest Reserve (MOUS-W) in Guinea and Kouadianikro Forest Reserve (KDNK-W) in Ivory Coast (Fig. 1, Table 1, Suppl. material 1: datafile 1). Sites were classified in the field as gallery forest (-G sites) or woodland (-W sites) based on proximity to a flowing river. At each woodland site, a 50 by 50 m plot was established, but at the gallery forests sites, 30 by 80 m plots were established instead because uniform vegetation did not extend for 50 m perpendicular to the flow of the river. In order to characterize the tree community of each plot, we measured tree girth at a height above the ground of approximately 1.4 m for all trees with a girth larger than 15 cm, and used these measurements to calculate basal areas (basal area = girth^2^/4*π*). Trees were categorized as ECM or non-ECM (Brundrett 2017) based on species identification by members of the team. The species identity and girth of all ECM trees was recorded in order to provide an accurate representation of the ECM tree community within each site. Non-ECM trees were not identified to species, but were treated as a pool. The initial grouping into gallery forest and woodland sites based on the presence of a stream was later reassessed based on ordination of the tree communities, as described under statistics below.

In each plot, soil sample locations were selected according to the protocol used in Tedersoo et al. (2014). Ten ECM trees were chosen in proportion to the relative abundance of ECM tree species in the plot, while ensuring that each species in the plot was represented at least once, and that all sampled trees were at least eight meters apart. At each selected tree, two soil samples were collected roughly one meter on either side of the stem using a small sterilized spade to collect the top 5 cm of soil. The two soil samples were pooled in a plastic bag and homogenized by hand for roughly ten seconds. A sub-sample of around 250 mg of soil was placed in a separate 2.0 ml tube containing 750 ml of field lysis and preservation buffer (Xpedition Soil/Fecal DNA miniprep, Zymo Research Corporation, Irvine, California, USA) and lysed in the field using a portable bead beater (TeraLyser, Zymo Research Corporation).

### DNA extraction, amplification and sequencing the soil fungal communities

Field lysed samples were returned to Uppsala University (Sweden) for DNA extraction using the Xpedition Soil/Fecal Prep kit following the manufacturer’s protocol. DNA concentration and integrity were verified by 0.8% agarose gel electrophoresis in 0.5% Tris Acetate-EDTA buffer (Sigma-Aldrich, St. Louis, Missouri, USA) stained with 1× GelRed (Biotium Inc., Hayward, California, USA). Approximately 1500 bases of the rDNA ITS and LSU regions were amplified from all soil DNA extracts using the primer set ITS1 (White et al. 1990) and LR5 (Hopple Jr and Vilgalys 1994) with Phusion High-Fidelity DNA polymerase (Thermo Fisher Scientific, Waltham, Massachusetts, USA). We ran a thermo-cycling protocol as follows: an initial denaturation at 95 °C for 10 min followed by 30 cycles of denaturation at 95 °C for 45 s, annealing at 58 °C for 45 s and elongation at 72 °C for 90 s, with a final elongation at 72 °C for 10 min. Both primers were indexed for multiplexing (Suppl. material 2: datafile 2). Each PCR run included a blank sample and a positive control with DNA extracted from a commercially purchased fruit body of *Agaricusbisporus*. PCR products from a total of 90 samples as well as controls were purified using Sera-Mag SpeedBeads (GE Healthcare, Life Science, Chicago, IL, USA) and quantified using Nanodrop 2000C (ThermoScientific, Waltham, USA) before pooling the samples at equimolar proportions together with samples from another unpublished study for sequencing at Uppsala Genome Center (Sweden) using two cells on a Sequel system (Pacific Biosciences, Menlo Park, CA, USA). Sequences were delivered to us as circular consensus sequence FASTQ files. Raw reads are available in the European Nucleotide Archive (samples ERS5551933–ERS5552022).

### Bioinformatic sequence analyses

Rather than applying the typical OTU clustering approach using a preselected sequence dissimilarity threshold to control both sequencing error and intraspecies variation, we used model-based approaches to address sequencing errors and intraspecies variation separately. We first generated denoised amplicon sequence variants (ASVs) in DADA2 (Callahan et al. 2016), where variation due to sequencing error is removed or reduced. We then grouped ASVs into phylogeny-based species hypotheses (SHs) using a Poisson tree process model (PTP; Zhang et al. 2013), based on a phylogenetic tree built from the reads.

Denoised ASVs were generated from the dataset using the procedure established in Kalsoom-Khan et al. (2020) after assessing general read quality and ensuring that the majority of reads fell within the expected length (1–2 kb). Raw sequence reads were filtered and trimmed using the tool cutadapt (version 1.18; Martin 2011) to demultiplex reads based on the forward and reverse barcodes, to keep only reads with both primers present, and to remove the actual primer sequences from the reads. Amplicons sequenced in reverse were reverse complemented before continuing the analyses. PacBio-type chimeras were detected and removed using cutadapt (version 2.3; Martin 2011). Reads were filtered using DADA2 (version 1.9.3; Callahan et al. 2016), discarding sequences with more than 3 expected errors as well as those with a length outside the range of 1200–1800 bases. Filtered sequences were then denoised using DADA2, with complete pooling to increase the detection of low-abundance ASVs, and an increased alignment band size of 32 (default 16) due to the tendency of PacBio sequences to include indels. Singleton ASVs are not included in DADA2 denoising output. *De novo* chimera detection and removal were also performed in DADA2, with a minimum parent overabundance of 3.5 (default 1.5) and allowing detection of chimeras with a single base difference from their parent sequence. After denoising and chimera removal, 1147 ASVs representing 3.6% of initial reads remained in the dataset (Suppl. material 5: Table S1). An ASV occurrence table was generated with read count for each ASV across samples.

The tool ITSx (version 1.1-beta; Bengtsson‐Palme et al. 2013) was used to identify the different regions (ITS1, ITS2 and the LSU gene) of the ribosomal rDNA within each ASV. Taxonomy was assigned to the ITS1 and ITS2 regions separately using SINTAX (Edgar 2016) in VSEARCH (version 2.10.4; Rognes et al. 2016) and the UNITE sequence database (release date 2019-02-02; Kõljalg et al. 2013).

In order to create a phylogenetic tree to assist in grouping ASVs into species hypotheses and identification of sequences without good ITS database matches, we utilized the highly conserved LSU region of each sequence. All LSU regions were aligned using MAFFT (version 7.402; Katoh and Standley 2013). Three accuracy-based algorithms (L, G and E) were tested within MAFFT. The alignment generated with the G algorithm was selected after visual inspection of all three alignments, because it was determined to most consistently align homologous regions. Alignments were visualized in Aliview (version 1.25; Larsson 2014), manually trimmed and checked prior to tree generation. To aid in taxonomy assignment and delineation of species hypotheses, a maximum likelihood tree was inferred using RAxML in the CIPRES portal (Miller et al. 2010). The maximum likelihood tree contained multiple issues including non-fungal lineages and possible chimeras. Class-level taxonomic assignments with a SINTAX confidence value of 0.8 or higher were added to the ASV name in the tree file using a customized script (Kalsoom Khan et al. 2020) in order to aid pruning of non-fungal lineages from the initial phylogenetic tree. A series of alignments and trees were generated by stepwise removal of ASVs that were determined to be non-fungal based on SINTAX taxonomy assignment and their placement in the tree. The non-fungal ASV_1147 was maintained in the alignment to serve as an outgroup. This procedure identified a total of 1,014 fungal ASVs in the dataset and the lowest-rank taxonomy assignment available (confidence value of 0.8 or higher) was amended to each ASV name (Suppl. material 3: datafile 3). Additionally, the SH Matching tool (development version) from the PlutoF platform (Kõljalg et al. 2019) was used to assign ASVs to UNITE species hypotheses (SH) when possible. ASVs were assigned to the narrowest UNITE SH whose inclusivity threshold they satisfied. ASV sequences are deposited in the European Nucleotide Archive (accession numbers LR993318-LR994464).

We used the Poisson-tree process (PTP) method to generate phylogenetic SHs based on branch length distribution in a ML tree based on the LSU region, including all fungal ASVs (Zhang et al. 2013) (Suppl. material 4: datafile 4). The ML tree, described above, was uploaded to the bPTP online server (https://species.h-its.org/ptp/) and run for 500,000 generations, with a range of burn-in rates being tested, before settling on 0.15 as the final burn-in rate. Convergence of MCMC chains was examined manually using Tracer (version 1.7.1; Rambaut et al. 2018). The resulting SHs were manually checked on a per-SH basis by aligning the ITS2 regions of the included ASVs to ensure they represent relevant units of similarity across ITS2 (>98%) and to detect possible chimeras across ASVs. An SH occurrence table across samples was calculated based on combined read counts for all remaining ASVs that mapped into an SH.

Functional guilds were assigned to SHs based on their taxonomic annotation using the FUNGuild database (Nguyen et al. 2016) via FUNGuildR (version 0.1.0; http://github.com/brendanf/FUNGuildR), where genus-level taxonomy generally allows for reasonably robust assignment of functional guild. For statistical analysis of the ECM community, all SHs that were assigned to the ectomycorrhizal guild with a confidence level of Probable or Highly Probable were included.

### Statistical analyses

The robustness of vegetation type site classification was tested using a non-metric multidimensional scaling (NMDS) ordination based on Bray-Curtis distances calculated on total basal area in m^2^/ha for each ECM species and the total basal area for all non-ECM trees at each site. NMDS was calculated using the metaMDS function from vegan (version 2.5.6; Oksanen et al. 2013), using stepacross distances for sites with no shared species and without the automatic community normalization procedures, which are intended for count-based analyses.

Mean annual temperature and precipitation for each site were extracted from 1970–2000 historical averages in 2.5-minute maps from WorldClim 2.1 (Fick and Hijmans 2017) using the RGDAL package (version 1.5-16; Bivand et al. 2020). Differences in climate variables, tree number and tree girth between vegetation types were tested using the non-parametric Wilcoxon-Mann-Whitney test, employing plot as a blocking variable for tests using individual trees as sampling units.

Species accumulation curves and asymptotic diversity estimates were calculated using the iNEXT package (version 2.0.20; Hsieh et al. 2016). Curves were calculated at three different spatial scales: for each soil sample based on the number of sequencing reads; for each sampling plot based on the number of sequencing reads and the number of soil samples; and for each vegetation type based on the number of sequencing reads, the number of soil samples and the number of sampling plots.

The SH occurrence table was normalized to relative read abundance within each sample. Variation in the total fungal communities and ECM fungal communities were visualized using unconstrained NMDS based on Bray-Curtis dissimilarities between relative read abundances of SHs. NMDS was calculated using metaMDS as above, with stepacross distances for the ECM communities, where high beta diversity led to some samples having no shared species. The number of dimensions in each NMDS was increased until the stress was below 0.2. Correlations between vegetation type, host species and plot identity and the community composition of the total fungal as well as the ECM fungal community composition were tested for significance in a series of three permutation tests (Anderson 2001; McArdle and Anderson 2001) using the dbrda and anova.cca functions in vegan (Oksanen et al. 2013) based again on Bray-Curtis dissimilarities between relative read abundances of SHs. The combination of dbrda and anova.cca gives identical results to the more commonly used adonis and adonis2 functions when used for the same models but also allow the test to be applied to the residuals of known effects and the use of stepacross distances. First, the existence of plot-level effects between the nine sampling plots was assessed by an unconstrained permutation test on a dbRDA with plot as the only explanatory variable. Then, differences between the two vegetation types were confirmed by permuting plots but keeping the samples within each plot together, with vegetation type as the only explanatory variable. Finally, differences between the fungal communities associated with different host trees were tested by using a dbRDA model with the host tree species as the explanatory variable, fit to the residuals of the model based on site, and tested by permuting samples within plots. All permutation tests used 9999 permutations.

All statistical analysis and resultant figure generation was conducted in R (version 3.6.3; R Core Team 2020). Plots were generated using ggplot2 version 3.3.1 (Wickham 2016). Arrangement of multiple subplots as well as calculation of confidence ellipses were done with ggpubr version 0.3.0 (Kassambara 2020). Labels inside plots were placed using ggrepel version 0.8.2 (Slowikowski 2020). Color palettes are from ColorBrewer v2.0 (Harrower and Brewer 2003) via the RColorBrewer package (Neuwirth 2014) and I Want Hue (Jacomy 2013) via the hues package (Baumgartner and Dinnage 2019).

## Results

### Ectomycorrhizal hosts of two vegetation types in the Guineo-Sudanian transition zone

**Figure 2.**
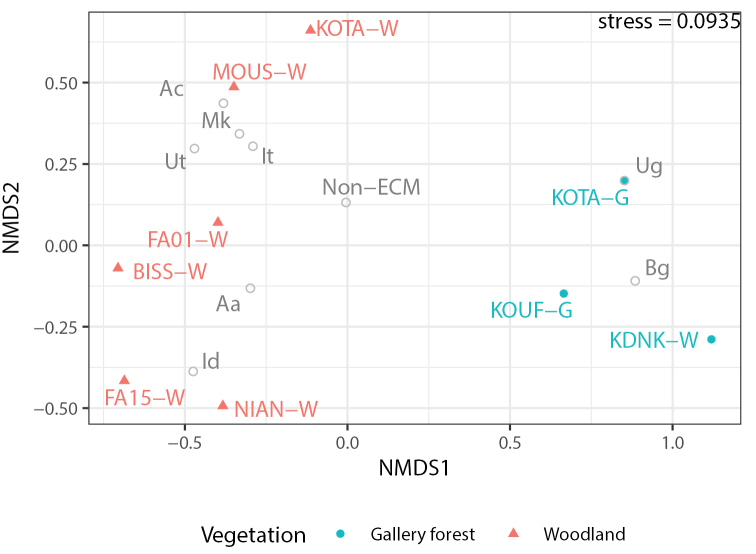
NMDS ordination of tree communities based on Bray-Curtis dissimilarities between sites, based on total basal areas of each ECM trees species separately and all non-ECM trees combined. The nine sites were classified into two distinct woodland types, woodlands in red and gallery forests in blue. Site abbreviations: Bissandougou Forest Reserve (BISS-W), Moussaya Forest Reserve (MOUS-W), Kota Waterfall (KOTA-G and KOTA-W), Kouadianikro Forest Reserve (KDNK-W), Kou Forest Reserve (KOUF-G), Niangoloko Forest Reserve (NIAN-W) and Farako Forest Reserve (FA01-W and FA15-W). ECM tree species abbreviations: Afzeliaafricana (Aa), Ac: Anthonothacrassifolia (Ac), Bg: Berliniagrandiflora (Bg), Id: Isoberliniadoka (Id), I.tomentosa (It), Monoteskerstingii (Mk), Uapacaguineensis (Ug) and Uapacatogoensis (Ut).

In order to verify our initial visual classifications of sites into gallery forests and woodlands, sites were condensed using the basal area of ECM tree species separately and all non-ECM trees combined. This classified the nine sites into two distinct vegetation types, gallery forest and woodlands, for further analysis in this study (Figs 1, 2). Three sites were classified as gallery forests in accordance with the initial classification of both Kota (KOTA-G) and Kou (KOUF-G), while Kouadianikro (KDNK-W) was initially classified as a woodland. Despite the location of Kouadianikro, several hundred meters from the nearest river on a sloping hillside, the closed canopy and high dominance of large *Berliniagrandiflora* trees at the site (100% of ECM trees, and 94% of all tree basal area) rendered it the most similar to the gallery forest sites, in particular KOUF-G. *B.grandiflora* was the most common ECM host tree at all three gallery forest sites (Table 1).

While all ECM host trees were *B.grandiflora* at Kou and Kouadianikro, three host species were present at Kota-G, with *Uapacaguineensis* being co-dominant with *B.grandiflora*. Across the gallery forest sites, the total basal area was on average 28.2 m^2^/ha, with ECM hosts making up 46–76% of total basal area (Table 1). The other six sites were classified as woodland, featuring a greater variation of host trees between sites and characterized by an open canopy and an average total basal area of 12.7 m^2^/ha. All woodland sites had more than one ECM host tree species, with dominant hosts including *Isoberliniatomentosa*, *Isoberliniadoka* and *Uapacatogoensis* (Table 1). Less abundant ECM host tree species encountered at these sites include *Monoteskerstingii*, *Anthonothacrassifolia* and *Afzeliaafricana*.

**Table 1.** Site characteristics.

Vegetation type and site	Country	Lat./Lon.	Elev (m)	MAT (°C)	MAP (mm)	All trees		Av. Girth		% ECM		ECM trees		Dom. ECM tree spp. (Rel abund)
						BA (m²/ha)	n	ECM (cm)	non (cm)	BA	Nr.	n	Sp	
**Gallery forest**			**375**	**26.7**	**1070**	**28.2**	**110**	**99**	**49**	**83**%	**59**%	**56**	**2**	
Kota-G	Benin	10°12.76"N, 1°26.77"E	500	26.5	1190	35.9	107	110	56	80%	56%	60	3	*B.grandiflora* (51%), *U.guineensis* (48%)
Kou	Burkina Faso	11°11.25"N, 4°26.48"W	375	27.4	980	26.4	113	86	48	77%	46%	54	1	*B.grandiflora* (100%)
Kouadianikro	Ivory Coast	7°37.77"N, 4°44.81"W	250	26.3	1030	22.3	72	102	42	94%	76%	53	1	*B.grandiflora* (100%)
**Woodland**			**440**	**26.4**	**1240**	**12.7**	**160**	**58**	**32**	**56**%	**40**%	**65**	**3**	
Bissandougou	Guinea	10°11.33"N, 9°11.60"W	425	25.9	1520	8.3	205	29	34	59%	62%	125	4	*U.togoensis* (54%), *I.doka* (43%)
Moussaya	Guinea	10°42.24"N, 9°59.71"W	430	25.8	1460	14.3	297	39	26	51%	37%	115	3	*U.togoensis* (71%)
Farako 01	Mali	11°14.12"N, 5°25.25"W	460	26.7	1080	9.1	58	91	37	46%	35%	15	2	*I.tomentosa* (70%)
Farako 15	Mali	11°14.38"N, 5°25.15"W	460	26.7	1080	8.4	121	63	17	66%	42%	20	3	*I.doka* (52%), *I.tomentosa* (40%)
Kota-W	Benin	10°12.54"N, 1°26.73"E	515	26.5	1190	18.1	200	60	45	44%	27%	54	3	*I.tomentosa* (86%)
Niangoloko	Burkina Faso	10°10.33"N, 4°55.74"W	345	27.0	1140	18.0	168	67	32	69%	38%	60	3	*I.doka* (89%)

Abbreviations: Lat./Lon.: Latitude and longitude; Elev: Elevation; MAT: Mean annual temperature; MAP: Mean annual precipitation; Tot. BA: Total basal area for all trees with girth larger than 15 cm; % ECM: Percentage of trees (girth > 15 cm) belonging to ectomycorrhizal (ECM) host species, by total basal area (BA) and total number (Nr); Av. Girth: Average girth of trees, separated into ECM host species (ECM) and other species (non); ECM trees: number of ECM host trees present (n), and number of species represented (Sp); Dom. ECM tree spp.: (co-)dominant ECM host species, as fraction of all ECM host trees. Numbers in bold are means for each vegetation type across sites.

There were no statistically significant differences between the two vegetation types in elevation (Z = -0.77, p = 0.44), mean annual temperature (Z = 0.39, p = 0.70) or mean annual precipitation (Z = -1.4, p = 0.15). The average girth of ECM host trees was generally larger than that of non-ECM trees in both gallery forests (Z = 6.17, p = 8.2e-7) and woodlands (Z = 10.025, p < 2.2e-16). Trees in gallery forests tended to have greater girth than those in woodlands for both ECM host trees (Z = 4.93, p = 8.7e-12) and non-ECM trees (Z = 2.68, p = 0.0073). The total basal area of all trees (Z = 2.32, p = 0.020) and the fraction of the total basal area represented by ECM trees (Z = 2.32, p = 0.020) were both greater in gallery forest plots than woodland plots. There was no significant difference in the total number of trees (Z = -1.55, p = 0.12), or the number of ECM trees (Z = -0.39, p-value = 0.70) between vegetation types (Table 1).

### Soil fungal communities in gallery forests and woodlands

**Figure 3.**
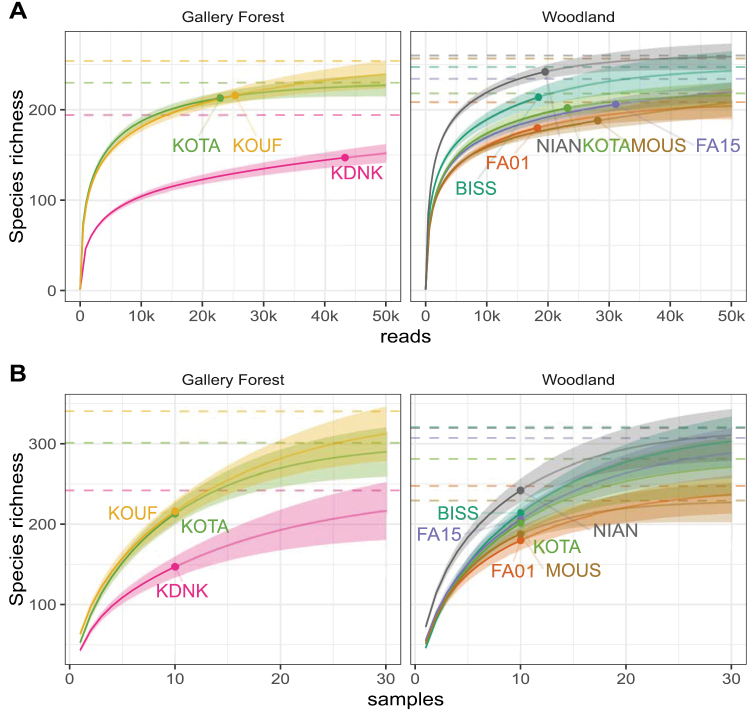
Species accumulation curves for each plot. Curves are based on SHs, by sequencing depth (**A**) and number of trees sampled (**B**), presented separately for three gallery forest sites (left panels) and six woodland sites (right panels). Points represent the observed species richness at the actual sequencing depth and trees sampled in **A, B** respectively. Thin lines represent the accumulation curve calculated by rarefaction (darker) and extrapolation (lighter); shaded regions represent the associated 95% confidence intervals. Dotted lines represent the asymptotic estimate for each site.

Across the nine plots, a total of 520 soil fungal taxa were detected as SHs based on branch length distribution in a ML tree generated from an alignment of the LSU region of 1,014 fungal ASVs (Suppl. material 3: datafile 3). Almost 70% of these were represented by only one ASV, together accounting for 17.5% of all reads. Species accumulation curves for the nine plots demonstrate that sampling was close to saturation with regard to sequencing depth (Fig. 3A). While more species would have been detected in each sample had we sequenced more (Suppl. material 5: Fig. S1), this is not predicted to translate into higher richness detected per site. On the other hand, species accumulation curves based on the number of samples were nowhere near saturated (Fig. 3B). These results indicate that we would have recovered more taxa per site if we had sampled 30 instead of 10 trees at each site. Estimated species richness from read-based species accumulation curves ranged from close to 200 to 250 soil fungal taxa per site (Fig. 3A) and does not appear to differ between the two vegetation types. Species richness estimated per vegetation type suggest an overall higher species richness in gallery forests compared to woodlands, however, confidence intervals for the estimates overlap (Suppl. material 5: Fig. S2).

**Figure 4.**
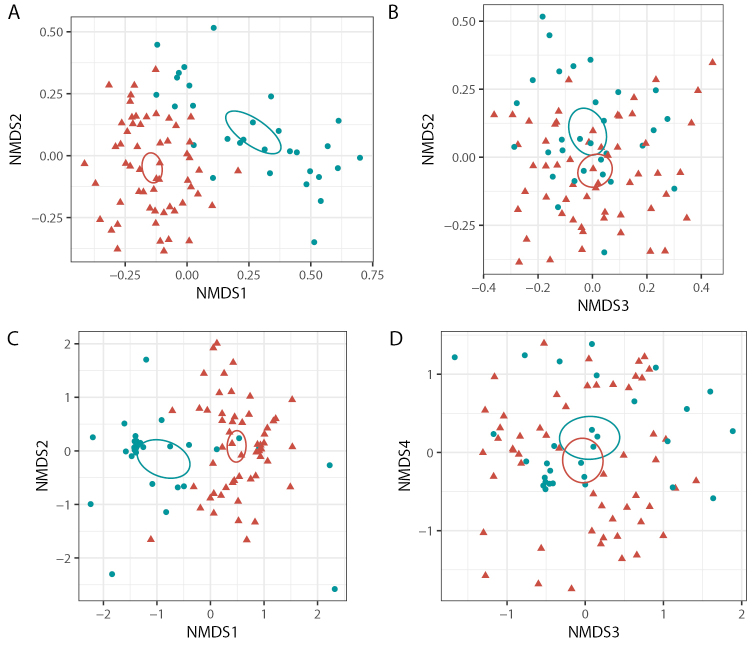
NMDS ordination of fungal communities based on Bray-Curtis dissimilarity of species hypothesis-based community composition, grouped into woodland (W) and gallery forests (GF) samples, for All fungi, axis 1–2 (**A**) and axis 2–3 (**B**), and for ECM fungi, axis 1–2 (**C**) and axis 3–4 (**D**). Stress value = 0.1902 for all fungi and 0.1723 for ECM fungi. Ellipses represent 95% confidence intervals around the mean of each vegetation type.

Ordination analysis based on relative abundance of soil fungal SHs show that community structure is affected by vegetation type (Fig. 4) and host species (Suppl. materials 5: Fig. S3, Table S2). Permutation tests confirmed the effect of vegetation type (P=0.012 for both ECM and total fungal communities). However, within plots, the effect of host species is not significant (P=0.44 and P=0.040 for ECM and total soil fungi respectively). At plot level, ECM host community composition is likely more important for shaping local fungal communities relative to the closest host tree sampled.

### Soil fungal communities in gallery forests and woodlands

**Figure 5.**
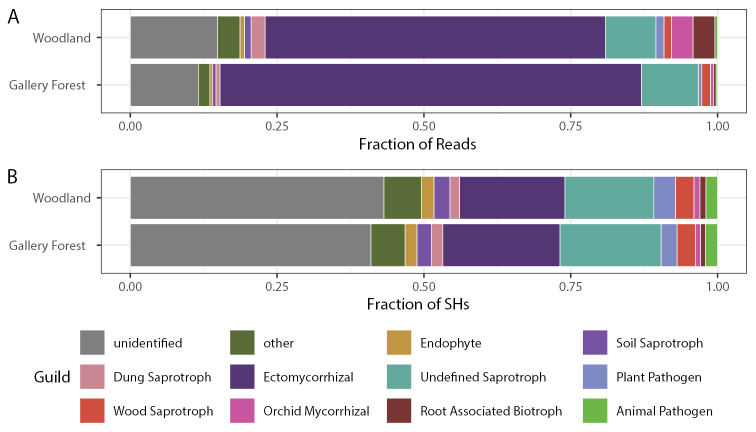
Fungal guild assignment of the soil fungal community in gallery forest and woodlands. Abundance measured as fraction of reads (**A**) and richness measured as fraction of species hypotheses (SH) (**B**) Guilds representing less than 2% of both abundance and richness are grouped together in “other”.

After evaluating the taxonomic affiliation of all ASVs based on their phylogenetic placement in the tree (Suppl. material 3: datafile 3), class-level taxonomy was assigned to 83% of the SHs, together representing 97% of the fungal reads in both vegetation types (Suppl. material 5: Fig. S5). The most abundant group was by far the Agaricomycetes, representing 71% of the reads in woodlands and 84% in gallery forests. Agaricomycetes was also the most species-rich class; with 174 taxa it represented 33% of the detected SHs in both vegetation types. The second most species-rich class was the Dothideomycetes, with 103 species, encompassing only 5.3% of the reads across all samples (Suppl. material 5: Fig. S5). In woodlands, 85% of the reads could be assigned to a fungal guild, and for gallery forest the corresponding number was 88% (Fig. 5A). The vast majority of guild-assigned reads were identified as ECM fungi: 58% in woodlands and 72% in gallery forests. Across both vegetation types, 57% of the SHs were assigned to a fungal guild, with 17% of the SHs representing ECM fungi (Fig. 5B).

**Figure 6.**
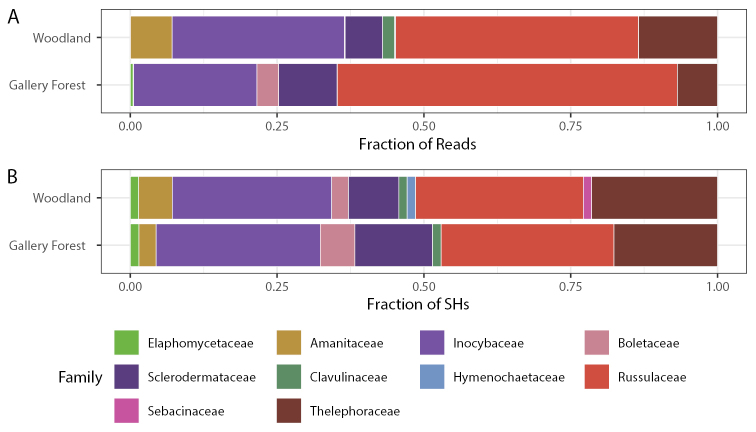
Taxonomic composition to family level of ECM fungi in gallery forest and woodlands. Abundance measured as fraction of reads (**A**) and richness measured as fraction of species hypotheses (SH) (**B**).

Except for two SHs in the family Elaphomycetaceae (Ascomycota), the ECM fungal communities in both vegetation types are made up of species in the phylum Basidiomycota (Fig. 6), and nine ECM lineages were identified to family level in that phylum. Based on both abundance and richness, the Russulaceae and Inocybaceae dominate the ECM fungal soil communities across both vegetation types (Fig. 6). A total of 29 SHs in Russulaceae were detected in both vegetation types, but the family Russulaceae was more abundant in gallery forests, accounting for 58% of the reads assigned to ECM, compared to 41% in woodlands (Fig. 6A). The families Thelephoraceae, Amanitaceae, Clavulinaceae and Hymenochaetaceae were all more abundant and more species-rich in woodlands compared to gallery forest. The families Boletaceae and Sclerodermataceae, on the other hand, were more abundant and diverse in gallery forest (Fig. 6). Sebacinaceae accounted for an average of 11% of reads and 3.3% of SHs in woodland sites, but only 1.4% of reads and 2.5% of SHs in gallery forest. However, of these only a few rare SHs, all in woodlands, were assigned to guild as probable ECM. Guild assignments are listed along with accession numbers and taxonomy assignment in Suppl. material 3: datafile 3.

The overall ordination patterns of the ECM communities are similar to those of the total soil fungal community (Fig. 4, Suppl. material 5: Figs S3, S4), and ECM communities are significantly affected by vegetation type (Fig. 4C). Interestingly, the species of ECM host tree located closest to each sample does not significantly (p=0.44) affect the ECM fungal community composition once site-level effects are excluded (Suppl. material 5: Fig. S3C, D). However, *B.grandiflora* seems to have the most distinct fungal communities, especially when it comes to the ECM fungal community (Suppl. material 5: Fig. S3C).

## Discussion

Most tropical tree species form symbiotic interactions with arbuscular mycorrhizal fungi (Smith and Read 2010). The fraction of tropical trees that instead interact with ectomycorrhizal fungi commonly form characteristic monodominant stands (Corrales et al. 2018). Indeed, ECM host trees make up 77–94% of the basal area in gallery forest plots of our study, and are dominated by *B.grandiflora* that is the only ECM host species in two of the three gallery forest plots (Table 1). The woodlands are an exception to this pattern, where monodominant stands are not observed (Lykke and Sambou 1998; Kakaï and Sinsin 2009; Houdanon et al. 2019). Instead, ECM host trees made up on average 56% of the basal area in these plots with two to four different host species in each plot (Table 1).

The total fungal community as well as the ECM fungal communities in gallery forest soils are different from those in woodland soils (Fig. 4). This effect is likely driven by the dominance of *B.grandiflora* which is the only host tree species that appears to have a distinct soil fungal community based on our ordination analysis (Suppl. material 5: Fig. S3A,C). However, abiotic factors such as soil moisture and soil chemistry were not determined in the present study and could also explain at least part of the observed differences. The overall species richness is not different between the vegetation types (Fig. 3). The gallery forest site Kouadianikro stands out with the lowest estimated richness of all sites. This is also the site from which we generated the highest number of sequences, although the number of samples collected, rather than the sequencing depth, appears to limit species detection in our study.

Spatial effects influence beta diversity of ECM fungi, more so in tropical ecosystems than in boreal forests (Bahram et al. 2013). The patchy distribution of ECM-dominated stands in tropical woodlands and forests has been suggested as one explanation for this observation, but other drivers include different soil characteristics, altitude and host specificity (Corrales et al. 2018). We also captured high beta diversity, especially for the ECM fungal community, in both vegetation types. In our survey, we sampled woodland more intensely than gallery forests. Even so, the species accumulation curves indicate that we would have needed to sample at least twice as many sites for our sampling to reach saturation.

Our data largely confirms earlier observations that the ECM fungal communities of West Africa are dominated by fungi in the families Russulaceae and Thelephoraceae (Bâ et al. 2012; Tedersoo and Smith 2013, 2017). In our data, Inocybaceae is the second most abundant and species-rich ECM lineage after Russulaceae. While both of these families contain predominantly fruiting body-forming fungi (De Kesel et al. 2002; Yorou and De Kesel 2011), many SHs could not be identified even to genus level. Assignment of ECM status in Sebacinales based on taxonomy is problematic due to the presence of multiple mycorrhizal types and recent taxonomic changes in the order (Weiss et al. 2004; Garnica et al. 2016), which have not been uniformly propagated into database annotations. However, based on their abundance in ECM woodlands, it is probable that at least some of the detected Sebacinaceae taxa are, in fact, ECM. Many fungal species in West Africa lack reference sequences and our dataset thus provides a mycological resource for future analysis of both described and hitherto undescribed or at least unsequenced fungal species of ECM-dominated woodlands and gallery forests in the Guineo-Sudanian transition zone.

Based on fruiting body inventories in Benin, Yorou and De Kesel (2011) demonstrated that gallery forests dominated by ECM trees represent unique, ECM species-rich habitats in West Africa. In the present study, soil fungal communities from gallery forests were enriched in Boletaceae relative to woodland sites. This corroborates the findings of Yorou and De Kesel (2011) that the Kota gallery forest (including sample site KOTA-G) features the most diverse assemblage of Boletaceae in Benin. In contrast, the lack of sequences from *Cantharellus* (Hydnaceae, Cantharellales) in the present study is striking, and likely due to primer mismatches (Tedersoo et al. 2015). In the Guinean and Sudanian ecozones, fruiting bodies of cantharelloid taxa are commonly found associated with gallery forests dominated by *B.grandiflora* and *U.guineensis* (De Kesel et al. 2011, 2016; Buyck et al. 2020). In Benin, gallery forests host a higher diversity of cantharelloid taxa compared to woodlands (Yorou and De Kesel 2011).

## Conclusion

Gallery forest and woodlands in the Guineo-Sudanian transition zone harbor partially overlapping and differently structured soil fungal communities. Site-specific composition of ECM host tree species shapes ECM fungal communities and total soil fungal communities. Our data provides a baseline, albeit incomplete, for phylogenetic placement and taxonomic resolution of environmental sequences from ECM-dominated forests in the Guineo-Sudanian transition zone. Sampling of more samples per site and more sites of the gallery forest is needed for a more complete characterization of the studied ecosystems.

## Supplementary materials

Supplementary material 1 Datafile 1 Data type Site and sample information Explanation note This file contains site and host tree information from ECM dominated woodlads of West Africa sampled during the National Geographic Society exploration grant #CP-126R-17. https://binary.pensoft.net/file/554300This dataset is made available under the Open Database License (http://opendatacommons.org/licenses/odbl/1.0/). The Open Database License (ODbL) is a license agreement intended to allow users to freely share, modify, and use this Dataset while maintaining this same freedom for others, provided that the original source and author(s) are credited.Peter Meidl, Brendan Furneaux, Kassim Tchan, Kerri Kluting, Martin Ryberg, Marie-Laure Guissou, Soro Bakary, Aïssata Traoré, Gbamon Konomou, Nourou Yorou, Anna Rosling

Supplementary material 2 Datafile 2 Data type Barcodes and primer sequences Explanation note This file contains the primer barcode sequence information for forward primer ITS1 and reverse primer LR5 used for amplification of total fungal (and other eukaryotes) from soil samples collected in ECM dominated woodlands of West Africa collected during the National Geographic Society explorer grant #CP-126R-17. https://binary.pensoft.net/file/554301This dataset is made available under the Open Database License (http://opendatacommons.org/licenses/odbl/1.0/). The Open Database License (ODbL) is a license agreement intended to allow users to freely share, modify, and use this Dataset while maintaining this same freedom for others, provided that the original source and author(s) are credited.Peter Meidl, Brendan Furneaux, Kassim Tchan, Kerri Kluting, Martin Ryberg, Marie-Laure Guissou, Bakary Soro, Aïssata Traoré, Gbamon Konomou, Nourou Yorou, Anna Rosling

Supplementary material 3 Datafile 3 Data type ASV list with accession nr, taxonomy and functional guild assignment Explanation note This file contains a list of all 1147 ASVs with accession nr and the taxonomy assignment inlcuding UNITE SH when assigned and functional guild assignment. Generated from soil samples collected in ECM dominated woodlands of West Africa collected during the National Geographic Society explorer grant #CP-126R-17. https://binary.pensoft.net/file/554302This dataset is made available under the Open Database License (http://opendatacommons.org/licenses/odbl/1.0/). The Open Database License (ODbL) is a license agreement intended to allow users to freely share, modify, and use this Dataset while maintaining this same freedom for others, provided that the original source and author(s) are credited.Peter Meidl, Brendan Furneaux, Kassim Tchan, Kerri Kluting, Martin Ryberg, Marie-Laure Guissou, Bakary Soro, Aïssata Traoré, Gbamon Konomou, Nourou Yorou, Anna Rosling

Supplementary material 4 Datafile 4 Data type Phylogenetic tree for inference of SHs Explanation note A Phylogenetic tree of all fungal ASVs in the study. https://binary.pensoft.net/file/554303This dataset is made available under the Open Database License (http://opendatacommons.org/licenses/odbl/1.0/). The Open Database License (ODbL) is a license agreement intended to allow users to freely share, modify, and use this Dataset while maintaining this same freedom for others, provided that the original source and author(s) are credited.Peter Meidl, Brendan Furneaux, Kassim Tchan, Kerri Kluting, Martin Ryberg, Marie-Laure Guissou, Bakary Soro, Aïssata Traoré, Gbamon Konomou, Nourou Yorou, Anna Rosling

Supplementary material 5 Table S1 and Figs S1–S4 Data type table and images Explanation note Supplementary tables and figures. https://binary.pensoft.net/file/554304This dataset is made available under the Open Database License (http://opendatacommons.org/licenses/odbl/1.0/). The Open Database License (ODbL) is a license agreement intended to allow users to freely share, modify, and use this Dataset while maintaining this same freedom for others, provided that the original source and author(s) are credited.Peter Meidl, Brendan Furneaux, Kassim Tchan, Kerri Kluting, Martin Ryberg, Marie-Laure Guissou, Bakary Soro, Aïssata Traoré, Gbamon Konomou, Nourou Yorou, Anna Rosling
