# Supplementary material for: Soil fungal communities of ectomycorrhizal dominated woodlands across West Africa
Source: MycoKeys. 2021 Jun 11;81:45–68. doi: 10.3897/mycokeys.81.66249 (PMC8390883; doi:10.3897/mycokeys.81.66249)
Supplement: Supplementary material 5 — Table S1 and Figs S1–S4 [file mycokeys-81-045-s005.docx]

**Supplementary information**

**Title:** Soil fungal communities of ectomycorrhizal dominated woodlands across West Africa

**Authors:** Peter Meidl^1*^, Brendan Furneaux^2*^, Kassim Tchan^3**^, Kerri Kluting^1**^, Martin Ryberg^2^, Marie-Laure Guissou^4^, Bakary Soro^5^, Aïssata Traoré^6^, Gbamon Konomou^7^, Nourou Yorou^3^, Anna Rosling^1***^

**List of content**

**Table S1.** Loss of reads and ASVs through bioinformatic processing steps

**Table S2**. Permutation tests of factors structuring soil fungal communities

**Figure S1.** Species accumulation curve based on sequencing depth in each sample.

**Figure S2.** Species accumulation curve by sequencing depth and trees sampled

**Figure S3.** Total fungal and ECM community ordination analysis per host tree

**Figure S4**. Total fungal and ECM community ordination analyses per site

**Figure S5.** Class level taxonomy of the characterized soil fungal community

**Supplementary datafile 1:** Sample and site info

**Supplementary datafile 2:** Primer sequence and sample barcodes

**Supplementary datafile 3:** ASVs, taxonomy and guild

**Supplementary datafile 4:** ML tree from ASV LSU alignment used to generate SH

**Table S1.** Loss of reads and ASVs through bioinformatic processing steps

| Step | Reads | Reads % | ASVs |
| --- | --- | --- | --- |
| Demultiplexing | 376,319 | 100% | - |
| Quality filtering | 293,360 | 78.0% | - |
| Denoising | 242,886 | 64.5% | 1,201 |
| Chimera filtering | 240,705 | 64.0% | 1,147 |
| Fungi only | 230,150 | 61.2% | 1,014 |

**Table S2.** Permutation tests of factors structuring soil fungal communities.

| Comm. | Factor | Cond. | Perm. | Within | R^2^ | F | Df | P |
| --- | --- | --- | --- | --- | --- | --- | --- | --- |
| All fungi | plot | - | samples | - | 0.189 | 2.27 | 8/78 | 0.0001 |
|  | veg. type | - | plots | - | 0.054 | 3.99 | 1/70 | 0.0117 |
|  | host sp. | plot | samples | plots | 0.081 | 1.08 | 7/71 | 0.4534 |
| ECM | plot | - | samples | - | 0.344 | 4.60 | 8/70 | 0.0001 |
|  | veg. type | - | plots | - | 0.149 | 10.69 | 1/61 | 0.0117 |
|  | host sp. | plot | samples | plots | 0.087 | 1.39 | 7/63 | 0.4398 |

For each test, the effect of “Factor” was tested, conditional on the effect of “Cond.”. Sampling units as listed in “Perm.” were permuted, but constrained to remain within the unit listed in “Within”. Given R^2^ values are based on the total variance; i.e. for the model of total fungal community based on host species conditional on plot, plot explains 0.189 of the total variance, host species explains an additional 0.081, and the remaining 0.730 is residual variance. F values and model/residual degrees of freedom are given for completeness, but the P-values are based on 9999 permutations, not on the χ^2^ distribution with the given degrees of freedom.


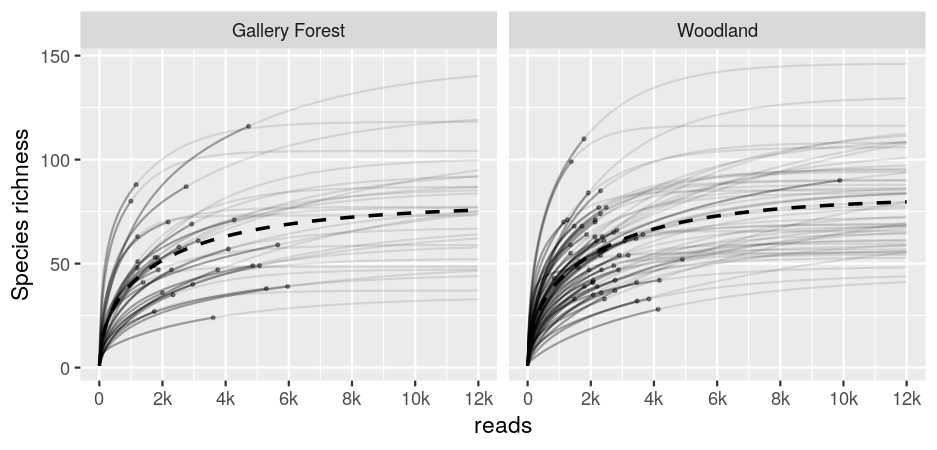

**Figure S1. Species accumulation curves per sample**

Species accumulation curve based on sequencing depth in each sample. Points represent the actual sequencing depth and observed species richness.  Thin lines represent the species accumulation curve based on rarefaction (darker) and extrapolation (lighter). The thick dashed line is the mean of all samples in each vegetation type.


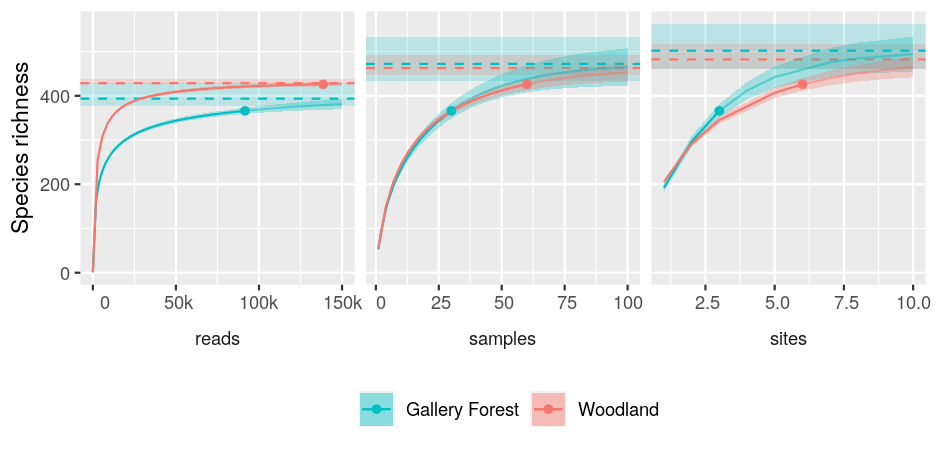


**Figure S2. Species accumulation curves per vegetation type**

Species accumulation curve for each vegetation type, based on sequencing depth (left), number of trees sampled (center) and number of sites (right). Points represent the actual sampling depth or sample number, and the observed species richness. Thin lines represent the accumulation curve calculated by rarefaction (darker) and extrapolation (lighter); shaded regions represent the associated 95% confidence intervals. Dotted lines represent the asymptotic estimate for each site, with 95% confidence interval as a shaded region.


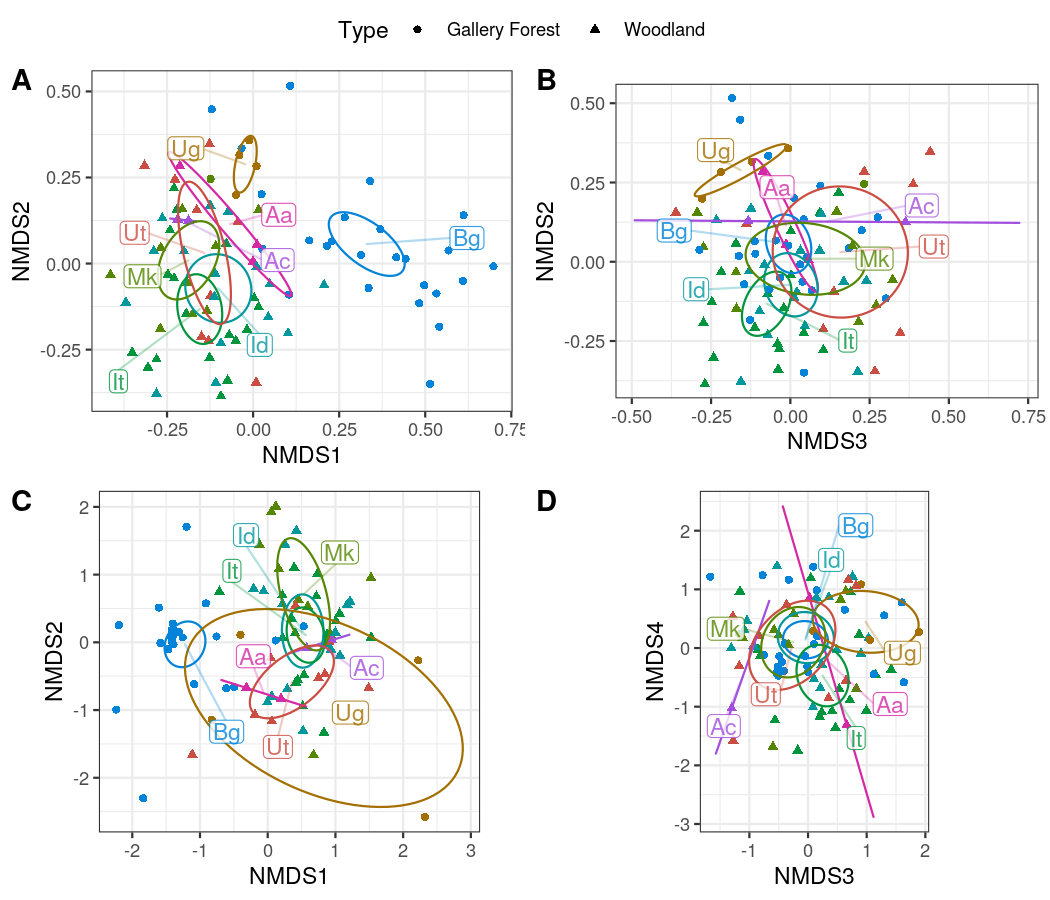


**Figure S3. Total fungal and ECM community ordination analysis by host species**

NMDS ordinations of species hypothesis based community composition for all species hypotheses by sampled host ECM tree, for all soil fungi axes 1–2 (A) and 2–3 (B); and for ECM fungi only, axes 1–2 (C) and 3–4 (D). Stress value = 0.1902 for all soil fungi and 0.1723 for ECM fungi. Host tree species abbreviations: Aa: *Afzelia africana*, Ac: *Anthonotha crassipes*, Bg: *Berlinia grandiflora*, Id: *Isoberlinia doka*, It: *Isoberlinia tomentosa*, Mk: *Monotes kerstingii*, Ug: *Uapaca guineensis*, Ut: *Uapaca togoensis*.


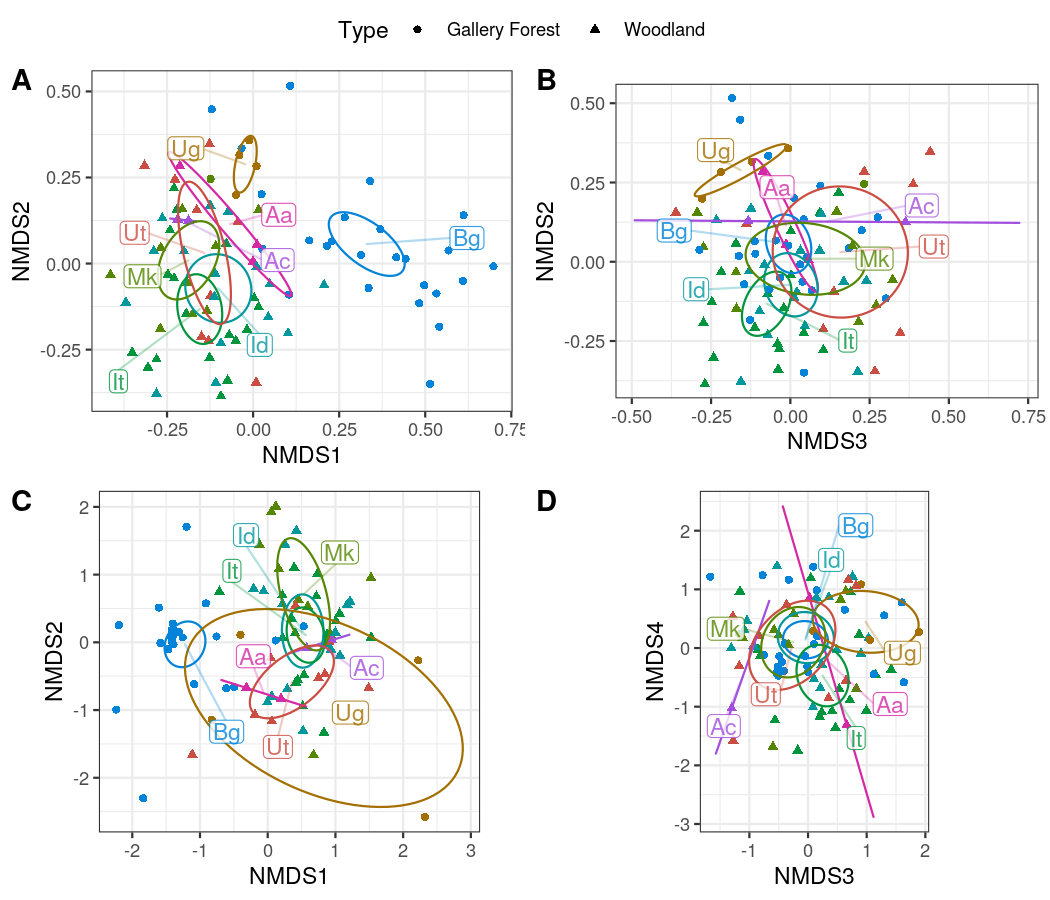


**Figure S4. Total fungal and ECM community ordination analysis by site**

NMDS ordinations of species hypotheses based community composition, by sampled host ECM tree, for all soil fungi, axes 1–2 (A) and 2–3 (B); and for ECM fungi only, axes 1–2 (C) and 3–4 (D). Stress value = 0.1902 for all soil fungi and 0.1723 for ECM fungi. Host tree species abbreviations: Aa: *Afzelia africana*, Ac: *Anthonotha crassipes*, Bg: *Berlinia grandiflora*, Id: *Isoberlinia doka*, It: *Isoberlinia tomentosa*, Mk: *Monotes kerstingii*, Ug: *Uapaca guineensis*, Ut: *Uapaca togoensis*.


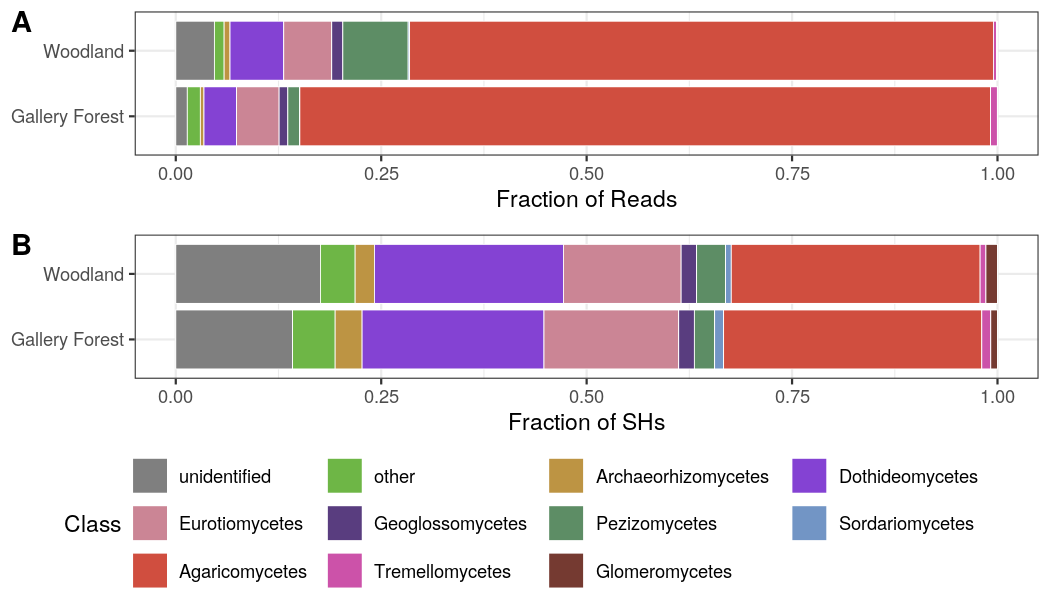


**Figure S5. Class level taxonomy**

Class level taxonomy of the characterized soil fungal community of gallery forest and woodlands. Average abundance across plots, calculated as (A) fraction of reads and (B) richness measured as fraction of species hypothesis (SH). Classes representing less than 1% in either abundance or richness are grouped together as “other”.
